# Editorial Notes

**Published:** 1941

**Authors:** 


### Editorial Notes

**American Ambulance, Great Britain.**

STATIONED in every Regional area of Great Britain, the large blue-grey cars of the American Ambulance, Great Britain, bearing in a circle the crossed flags of Britain and the U.S.A., have now become a familiar sight. The service is another of America's gestures to Britain in our hour of need. Entirely financed and maintained by American donations, it is operated under the direction of the Ministry of Health.

Behind the foundation of the American Ambulance, Great Britain, with its total strength of 260 motorized ambulances surgical units and mobile first-aid posts, there lies an interesting story.

In the spring of 1940 the American Society in London met to decide on the nature of the celebration to be held by American residents here to commemorate the 4th of July, American Independence Day. For forty-two years American residents here who formed the Society have marked their day of independence with a banquet and ball.

This time, however, a gentleman of medium stature, Herculean shoulders, and hair *á la brosse*, got up at the meeting. He had a suggestion to make. It was :

That to his mind anything in the nature of a celebration at this period would be out of place, for although the 4th July day was a day set aside by Americans to commemorate Independence Day, it also unfortunately marked a war between America and Britain. Thus the thing to do, he thought, was for the Americans who had enjoyed the hospitality of Great Britain was not to celebrate but to make a worth-while gesture of assistance to their foster-country. The American who made this suggestion was Mr. Gilbert H. Carr, twice chairman of the American Society in London and at present Honorary Secretary. From his suggestion grew the American Ambulance, Great Britain, with its 260 motorized ambulances, surgical units and mobile first-aid posts, for the hurried transportation of complete surgical units to areas where they are most required, and four-stretcher ambulances for the transportation of war victims. In twelve months its 264 motor ambulances, surgical units and mobile first-aid posts have covered 1,728,992 miles and carried 71,511 patients.

Now listen to what the Minister of Health had to say when the American Ambulance, Great Britain, was turned over to him: "It is only a few weeks since I was informed that American residents in this country wished to present a number of ambulances for the services of civilian casualties through whatever ordeal lay ahead of the people of Britain. Needless to say, I accepted the suggestion with alacrity. But I did not think then that the offer, which was made with great modesty, would materialize so swiftly into such a magnificent gift as it has now become."

Mr. Gilbert H. Carr is now Director-General of the American Ambulance, Great Britain, but this is only one of this American friend of Britain's activities. In fact, he is probably the hardest working American in Britain's cause in this country. He is Honorary Secretary of the American Committee for Air Raid Relief and Honorary Secretary of the Advisory Committee of the British War Relief Society of America.

He seems, however, to have been born with a desire to help Britain. He was born in New York fifty-four years ago; he ran away at the age of fourteen to join the British Army in the Boer War. Two years later he returned to his schooling in New York City. In 1917 a younger Gilbert Carr hurried to the recruiting office when America entered the war, and was one of the first officers of the United States Army Expeditionary Force to land in this country.

The last Great War over, Gilbert Carr did not seem able to forsake his love for Britain, and he came back in a new guise, as a business-man to introduce Californian raisins to the British palate. Although competing for the first time with his British business cousins, he was so successful and well liked by them that he was twice made Chairman of the Incorporated Sales Managers Association of Great Britain, and is at present President of the London branch.

As Director-General of the American Ambulance, Great Britain, he is fortunate in having the experienced assistance of Mr. C. Glidden Osborne, the Director of Operations. Mr. Osborne was Director of Transport in France of the American Red Cross Ambulance in the last war.

On May 6th Mr. Gilbert Carr visited Bristol, and in company with the Lord Mayor (Alderman F. H. Underwood) inspected the American Ambulances operating in this city. He paid a glowing tribute to the local staff's efficiency, saying: "They are doing a splendid job." The following brief summary will give some idea of the kind of work these ambulances and cars are doing in Bristol:—

Evacuation of hospitals, and transfer of patients from one hospital to another, whether in Bristol or to hospitals in the South of England.

Transport of patients being admitted to hospital, to convalescent homes or to their own homes on recovery.

Transport of Ministry of Health stores in emergency to badly bombed areas.

Work in conjunction with Bristol A.R.P. services when possible.

The grey cars with their broad red line must be well known in Bristol and the neighbourhood. The 420 women drivers are all but two British, many of them were originally members of the F.A.N.Y. or of the Women's Mechanical Transport Corps. Some of them are veterans who drove in France in the last war, some of them were driving ambulances in France in this war, all of them are now embodied in the "American Ambulance, Great Britain."

Approximately £2,000 is spent weekly by the American Ambulance in maintaining the service in Britain. Americans in this country and in the United States have contributed £200,000 for the maintenance of the fleet of cars.

Although other aids that we receive from the United States may be of greater military importance, this effort of the American Ambulance, Great Britain, is more evident, more appreciable to the public. It is a grand way of celebrating American Independence Day, a day of victory which, it is whispered, Bristol never wholeheartily grudged to "the American Colonies."

**George Medal awarded to Two Bristol Nurses.**

Sister E. L. Stevens, Assistant Matron, and Sister V. E. A. Frampton, of the Bristol Maternity Hospital, Southwell Street, have been awarded the George Medal in circumstances described in the *London Gazette* as follows :—

"A message came to the hospital requesting assistance for a woman trapped under debris, who it was believed at the time was to give birth to a baby. Sister Stevens and Sister Frampton readily volunteered to go. The journey was made through a dangerous area. Seven persons were trapped and conditions were so dangerous that it was practically impossible to continue rescue operations. Sister Stevens was lowered into a narrow opening and found it necessary to lie flat to reach one casualty. She returned to the cellar, where her patient was lying entirely covered by debris. There was considerable danger as the building was liable to collapse. Despite this and in an atmosphere made poisonous by an escape of gas, the sisters carried on and after some hours the woman was released. Sister Stevens and Sister Frampton showed outstanding bravery and devotion to duty."

We congratulate the two sisters on this recognition of their brave work, and we are glad also to record that the victim of the incident has made a good recovery in hospital where she is still awaiting the birth of her baby.

**Matrons of the General Hospital and Children's Hospital.**

MISS A. C. ROBINS, Matron of the Bristol General Hospital, has been awarded the O.B.E. "for setting an outstanding example of fortitude and courage to her staff and for continuous devotion to duty under difficult conditions at the hospital." Miss G. R. Ellis, Matron of the Bristol Royal Hospital for Sick Women and Children has been awarded the M.B.E. for similar gallant leadership.

We are proud that these decorations conferred on their Matrons are a public recognition of the remarkable courage and endurance displayed by the whole nursing staffs of these two hospitals.

**John Wright & Sons Ltd.**

OUR sympathy is extended to Messrs. John Wright & Sons Ltd., the well-known Printers and Publishers of many medical works, whose premises, with the whole of their specialist plant and machinery were recently destroyed by enemy action. We are glad to know that they have been able to make arrangements to carry on their business from temporary offices at Gaunt House, 28 Orchard Street, Bristol. Premises and plant have been secured at Weston-super-Mare, where it is hoped production will be resumed very shortly. This old-established and experienced firm has a world-wide reputation for medical publications of which the city and the medical profession are justly proud. The *Medical Annual* for 1941 has been published as usual and the April issue of the *British Journal of Surgery*, which was completely destroyed, has been reprinted and circulated to subscribers.

**The late Professor F. E. Francis.**

DURING his short tenure of the office of Vice-Chancellor of the University, Professor Francis was responsible for an important change in the relations between the teaching hospitals and the University. No one was better acquainted with the medical staffs of the clinical institutions than Francis ; no negotiator could have been more friendly and welcome to the members of the medical staffs, for he had enjoyed the advantage of sharing for many years the house of one of the surgeons of the Infirmary, the late Harold Mole. In that hospitable bachelor establishment he had met most of us and learnt our foibles. This enabled him to draw up and have accepted the agreement by which the Clinical Teachers surrendered their right to pupils' fees, so that in

August, 1922, the Medical School became entirely incorporated with the University.

Thus the inter-regnum when Francis acted as Vice-Chancellor in 1921–22, is perhaps the most memorable chapter in the history of that office in the eyes of the Medical Faculty of the University.

His death is deeply regretted and our sympathy is offered to his widow.

